# Diagnostic Accuracy of Loop-mediated Isothermal Amplification Assay as a Field Molecular Tool for Rapid Mass Screening of Old World *Leishmania* Infections in Sand Flies and In Vitro Culture

**Published:** 2017

**Authors:** Mehdi GHODRATI, Adel SPOTIN, Teimour HAZRATIAN, Mahmoud MAHAMI-OSKOUEI, Ali BORDBAR, Sahar EBRAHIMI, Shirzad FALLAHI, Parviz PARVIZI

**Affiliations:** 1. Immunology Research Center, Tabriz University of Medical Sciences, Tabriz, Iran; 2. Dept. of Parasitology and Mycology, Faculty of Medicine, Tabriz University of Medical Sciences, Tabriz, Iran; 3. Molecular Systematics Laboratory, Parasitology Department, Pasteur Institute of Iran, Tehran, Iran; 4. Dept. of Parasitology and Mycology, Faculty of Medicine, Lorestan University of Medical Sciences, Khorramabad, Iran

**Keywords:** LAMP, PCR, *Leishmania*, Sandfly, Iran

## Abstract

**Background:**

We employed a highly sensitive loop-mediated isothermal amplification (LAMP) by targeting 18S rRNA gene to identify the rapid mass screening of *Leishmania* infections in captured sand flies of southwest Iran and In vitro culture.

**Methods:**

One hundred fifty sand flies were collected from 11 sites adjacent to Iraqi’s borders in southern parts of Khuzestan Province by using sticky sheets of paper and CDC miniature light traps during late May 2014 to Nov 2015. Following morphological identification of sand flies species, the DNA of infected samples was extracted and amplified by PCR and LAMP assays by targeting ITS-rDNA and 18S rRNA genes. The PCR amplicons were directly sequenced to conduct the phylogenetic analysis

**Results:**

Ten (6.6%) *Leishmania* infections were identified by LAMP assay (detection limit 0.01 parasites DNA) among infected *Sergentomyia baghdadis*, *S. sintoni* and *Phlebotomus papatasi* sand flies that was more sensitive than PCR (n=6.4%; (detection limit 10^1^ parasites DNA). LAMP can identify 10^1^–10^6^ promastigotes/100 μl RPMI 1640 while PCR recognized 10^4^–10^6^ promastigotes. The majority infection rate of sand flies was confirmed to *L. major* inferred by phylogenetic analysis.

**Conclusion:**

This is the first exploration characterized the Old World Leishmania infections by LAMP technique in both infected sand flies and In vitro conditions. The LAMP method because of its shorter reaction time, robustness, more sensitivity, lack of requirement of complicated equipment and visual discriminatory of positivity can be appeared a promising tool instead of PCR to identify low *Leishmania* loads and entomological monitoring of leishmaniasis in resource-limited endemic of the world.

## Introduction

Leishmaniases are neglected metazoonotic diseases which caused by an intracellular protozoan of the genus *Leishmania* in 98 endemic countries (1). Based on epidemiological importance, two etioparasitological agents including *L. major* and *L. tropica* have known as cutaneous leishmaniases (CL) in more than half of the Iranian provinces transmitted by biting of female infected sand flies of the genus *Phlebotomus* (2–4).

The average annual number of CL patients reported 18 884 (30.9–32/100 000 Iranian population) during 1983–2012 in Iran (5, 6) furthermore, the burden of leishmaniasis was estimated 95.34 and 4.16 yr for CL and visceral leishmaniasis (VL) respectively (7). Approximately 800 sand fly species have been explained that only some are medically considerable (8).

The identification and monitoring of natural *Leishmania* infection rate in sand flies are essential epidemiological indicators for estimation of transmitted risk, prevalence rate of disease and transmission intensity of infected sand flies. The recognition of natural infection of *Leishmania* parasite in sand flies is conventionally evaluated by parasite isolation from dissected sand flies and observation of their guts (9). However, this method is the most labor intensive and time-consuming, particularly when the amount of parasitic load is low among the endemic foci (10, 11). A number of *Leishmania*-DNA-based PCR techniques including restriction fragment length polymorphisms (RFLP), Multiplex-PCR, Nested-PCR, hybridization, sequencing and real-time PCR have been employed to characterize the natural infection rates in sand flies (12–16). Nevertheless, the conventional PCR assays take several hours and necessitate specialized equipment that makes their use impossible in under field conditions. In addition, the activity of Taq DNA polymerase is inhibited by tissue and blood components such as myoglobin, protoporphyrin, and immunoglobulin G (17–19).

Recently, loop-mediated isothermal amplification (LAMP) has been introduced as a rapid and sensitive alternative diagnostic technique compared to PCR in field conditions (20–22). The extensive applicability of LAMP has been established in the revealing of protozoan infections such as *Leishmania*, *Trypanosoma*, *Babesia,* and *Plasmodium* (20–22). In a similar study, the VL based on LAMP was evaluated and nested PCR assays using blood samples of infected dogs (23).

Some investigations have shown the importance of LAMP to identify vectors of infectious diseases (20–21). So far, a study has detected *Leishmania*-infected *Lutzomyia* in the New World by using the LAMP method (24).

The aim of this study was to conduct a highly sensitive LAMP assay in order to identify the rapid mass-screening of *Leishmania* infection in captured sand flies (In vivo) in Southwest Iran. As well, the analytical sensitivity of LAMP assay is optimized on identification of *Leishmania* in vitro culture.

## Materials and Methods

### Sandfly collection, study sites, and morphological identification

Sandfly spp. was captured from a large geographical scale in Khuzestan Province, where it is located southwest Iran border with Iraq country. One hundred fifty sand flies specimens were collected from 11 sites adjacent to Iraqi’s borders in southern parts of Khuzestan during their seasonal activity in late May 2014 to Nov 2015. Sticky sheets of paper and CDC miniature light traps were placed 1–1.5 m above the ground to sample the sand flies and were deployed before dusk until dawn. Sandfly traps were placed in gerbil burrow entrances, new developing urban areas, rural areas adjacent to the boundary lines and domestic animal shelters (25). All captured sand flies were examined and identified based on morphological characteristics of the head and abdominal Terminalia using compound microscopy (400×). The genitalia of each sandfly was carefully removed using micro-needles and slide-mounted in Berlese fluid following dissection using sterilized forceps.

### DNA extraction and Polymerase chain reaction amplification

The total DNA of the dissected thorax and anterior abdomen of infected sand flies was extracted using the modified method of Ish-Horowicz (14), GeNet Bio and a DynaBio^TM^ Kit (Bioneer Corporation, Seoul, Korea and Takapouzist Corporation, Tehran, Iran). PCR fragments were amplified as previously described (14). In addition, DNA samples used in this study were prepared from the following cultured *Leishmania* reference strain: *L.* (*Leishmania*) *major* (MHOM/SU/1973/5ASKH).

The PCR was used to detect *Leishmania* parasites in infected sand flies by targeting two genes; Internal transcribed spacer-ribosomal DNA (ITS-rDNA) about 480 bp and cyto-chrome b (Cyt *b*) about 880 bp. PCR products were subjected to electrophoresis in 1.5% aga-rose gel and were observed under ultraviolet light after staining for 15 min with (0.5 g/mL) safe stain.

### Loop-mediated isothermal amplification assay

The forward and backward external primers (F3 and B3) along with forwarding and backward internal primers (FIP and BIP) of 18S rRNA gene were employed to perform LAMP assay method (24). The primers had been designed using Primer Explorer version 4.0 software based on the conserved region of the *Leishmania* 18S rRNA marker. The employed LAMP primers are shown in Table 1.

**Table 1:** Oligo nucleotide sequences of 18S rRNA used for the LAMP assay

***Primers name***	***Sequence 5′→3′***
F3	GGGTGTTCTCCACTCCAGA
B3	CCATGGCAGTCCACTACAC
FIP	TACTGCCAGTGAAGGCATTGGTGGCAACCATCGTCGTGAG
BIP	TGCGAAAGCCGGCTTGTTCCCATCACCAGCTGATAGGGC

The LAMP assay was conducted in 25 μl of a reaction mixture consisting of 40 pmol/ul concentration of each inner primer (FIP and BIP), 5 pmol/ul concentration of each outer primer (F3 and B3), 8 U *Bst* 2.0 DNA polymerase (New England Biolabs), 1 μl SYBR Green I, 2.5 μl 10 X buffer, 1.4 mM of dNTPs, 3 mM of MgSo4, 0.8 M of Betaine and 1 μl of template DNA. The mixture was incubated at 64 °C for 60 min in a heating block and then heated at 80 °C to terminate the reaction. A positive control of *Leishmania* DNA and water as a negative sample was included in each LAMP assay. At the end of incubation, the presence of the target gene was characterized by the presence of white turbidity of magnesium pyrophosphate which detected visually by the naked eye. Accuracy of the findings was confirmed by both electrophoresis and fluorescence detection. LAMP products were electrophoresed on 1.5% agarose gel and were observed under ultraviolet light after staining by safe stain for 30 min. Positive samples showed the typical ladder pattern that was not a single band. For fluorescence detection, 1 μl of SYBR Green I was added on LAMP products and were irradiated with a UV lamp and photographed. The presence of fluorescence indicated the presence of the target gene. The analytical sensitivity of lAMP and PCR assays on infected sand flies was evaluated against 10-fold serial dilutions of *Leishmania* DNA (equivalent to 0.01 to 10^6^ parasites DNA). Additionally, to determine the analytical sensitivity of the LAMP and PCR tests on cultured parasites, 10-fold serial dilutions (10^1^–10^6^ parasites/100 μl RPMI 1640) of purified *L. major* was used as a template. The analytical specificity of LAMP primers on other parasitic diseases had previously described by (24). To re-confirm, the specificity of the LAMP primers was characterized by testing against DNA of non-leishmanial infections including; Cystic echinococcosis, malaria, toxoplasmosis, giardiasis, and cryptosporidiosis.

### DNA sequencing and phylogenetic analysis

To confirm the specificity of the LAMP primers, the amplicons were amplified and sequenced by targeting B3 and F3 outer primers. Individual sequences were aligned and edited at consensus positions use of Sequencher Tmv.4.1.4 Software for PC (Gene Codes Corporation). Additionally, to confirm the *Leishmania* spp. within the LAMP-positive sand flies, PCR products were amplified and sequenced with primers specific for *Leishmania Cyt* b. To evaluate the phylogenetic information provided by *Cyt* b marker, a Neighbor-Net network was built in SplitsTree 4.0 based on genetic distances calculated according to the Kimura-2 parameter model of nucleotide substitutions (26).

## Results

A total, 150 sand flies (24, 12, 6, 15, 19, 17, 17, 5, 7, 8 and 20 samples from Sarcheshmeh, Valayat, Arvan rood, Darkhovin, Sheneh, Maslavi, Seyedhasan, Abadan, Aboshanak, Arayez and Khoramshahr respectively) were individually screened for *Leishmania* infection (Table 2).

**Table 2:** Rapid mass-screening of *Leishmania* infection in sand flies from endemic foci of Khuzestan province, southwest Iran by LAMP and PCR assays

***Country/Province***	***Location***	***Sand fly species***	***No. examined***	***Molecular analysis***					
**Iran/Khuzestan**				PCR (ITS-rDNA and *Cyt* b)	LAMP (18s rRNA)
		*Ph. papatasi*	13	No. Infected (%)	No. Infected (%)
				0	1 (7.7)
	Sarcheshmeh	*S. sintoni*	5	0	0
		*S. iranica*	2	0	0
		*S. baghdadis*	4	0	0
	Valayat	*Ph. papatasi*	7	1(14.2)	2 (28.5)
		*Ph. alexandri*	5	0	0
	Arvan rood	*Ph. papatasi*	6	0	1(16.6)
	Darkhovin	*Ph. papatasi*	10	1(10)	1(0)
		*S. sintoni*	5	0	0
	Sheneh	S. *baghdadis*	14	1(7.1)	1(7.1)
		*Ph. papatasi*	5	0	1(20)
	Maslavi	*Ph .papatasi*	7	0	1(14.2)
		*Ph. alexandri*	3	0	0
		*S. sintoni*	7	1(14.2)	0
	Seyedhasan	*Ph. papatasi*	17	1(5.8)	2(11.7)
	Abadan	*Ph. papatasi*	5	0	0
	Aboshanak	*Ph. alexandri*	7	0	0
	Arayez	*Ph. papatasi*	8	0	0
	Khoramshahr	*ph. alexandri*	11	0	0
		*Ph. papatasi*	9	1(11.1)	0
	Total		150	6 (4.0)	10(6.6)

We identified five species among *Ph.* and *Sergentomyia* sand flies caught in 11 villages to the Khuzestan boundaries (Table 2). Two species of *Ph. genus*: *Ph. papatasi* and *Ph. alexandri* and three species of *Sergentomyia genus*: *S. sintoni*, *S. baghdadis* and *S. iranica* were found among collected sand flies (Table 2).

**Fig. 1:**
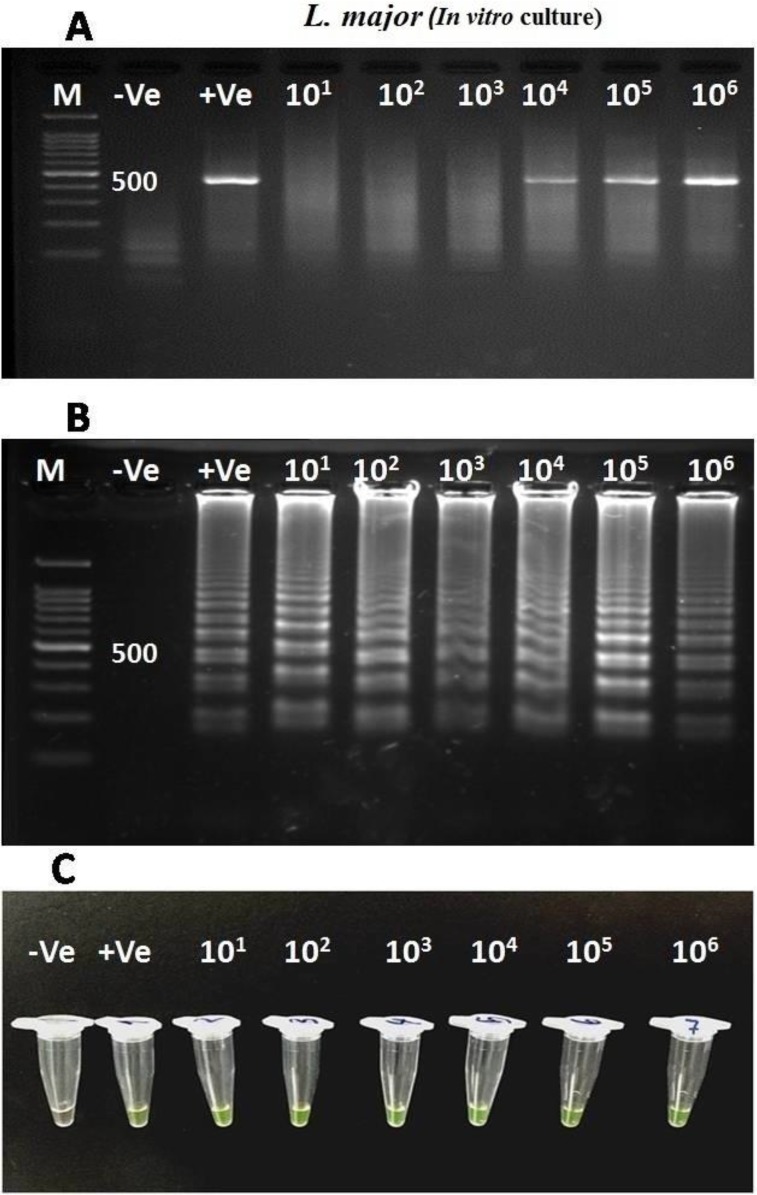
Sensitivity evaluation of LAMP and PCR assays using serial dilutions of *L. major* promastigotes in *In vitro* culture. (A) Single round-PCR by targeting ITS-rDNA gene (Amplified fragment; 480 bp). (B) LAMP, electrophoresis detection. (C) LAMP, visual detection by fluorescence. M=100 bp DNA ladder marker; +Ve: Positive control; −Ve: Negative control

Ten (6.6%) *Leishmania* infections were identified by LAMP assay in *Sergentomyia baghdadis, S. sintoni* and *Ph. papatasi* sand flies whilst, single round PCR was able to detect six (4%) infected sand flies (Table 2). The analytical sensitivity findings of In vitro culture showed that LAMP can identify 10^1^-10^6^ promastigotes/100 μl RPMI 1640 in *L. major* infections while PCR recognized 10^4^-10^6^ parasites by targeting ITS-rDNA gene (480 bp) (Fig. 1).

**Fig. 2:**
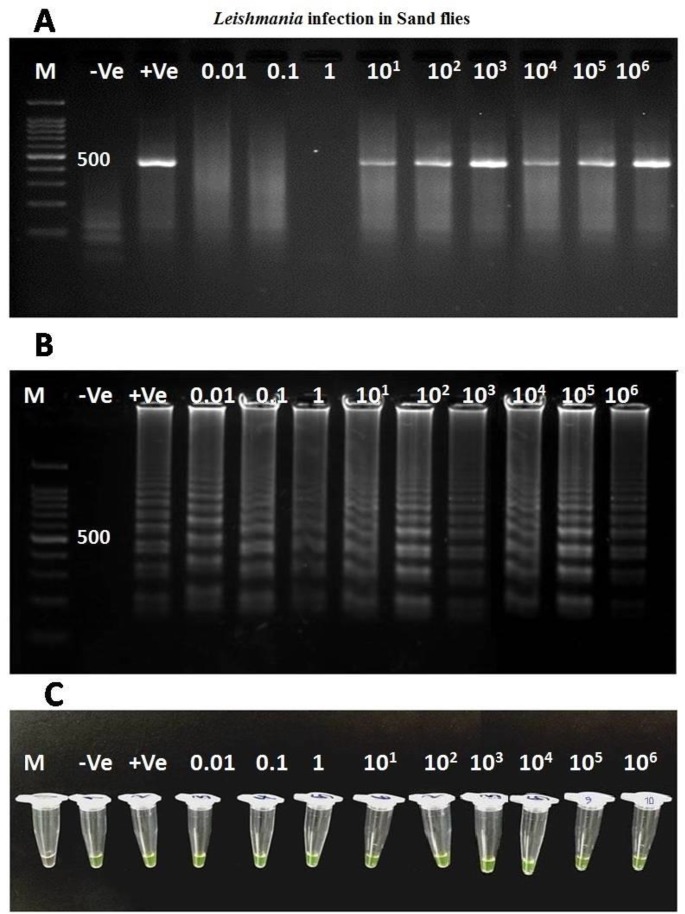
Sensitivity evaluation of LAMP and PCR assays using serial dilutions of *Leishmania* DNA in infected sand flies. (A) Single round-PCR by targeting ITS-rDNA gene (amplified fragment; 480 bp) (B) Agarose gel electrophoresis of LAMP products electrophoresis detection. (C) LAMP, visual detection by fluorescence. M=100 bp DNA ladder marker; +Ve: Positive control; −Ve: negative control

**Fig. 3:**
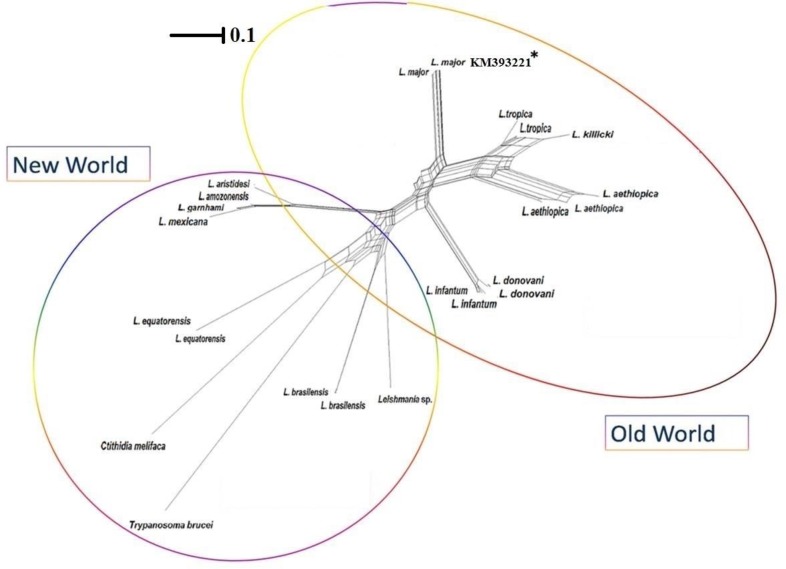
Neighbor-Net network according to *Cyt* b sequences of *Leishmania* spp. based on their geographical distribution in Old and New Worlds. The identified *L. major* (Accession no: KM393221*) in sand flies grouped at Old World complex. *Trypanoasoma brucei* was considered as an out-group branch in New World leishmaniasis

On the one hand, 0.01 *Leishmania* DNA is sufficient for LAMP detection in an infected sand fly whilst for PCR was optimized 10^1^ *Leishmania* DNA (Fig. 2). The value of sensitivity and specificity of LAMP for all infected sand flies were shown 100% and 100% respectively while these values for single round PCR were 60% and 100%. The LAMP products were sequenced by targeting outer primers (F3 and B3) of 18S rRNA gene where the *L. major* was definitely identified among all infected sand flies. To reconfirm the *Leishmania* spp. within LAMP-positive sand flies, *Cyt* b and ITS-rDNA genes were successfully amplified and sequenced. The topology of constructed phylogenetic tree is shown that the identified *L. major Cyt* b gene (Accession no. KM393221) supported in *L. major* clad at Old World leishmaniasis close to *L. tropica* complex (*L. ropica*, *L. killicki* and *L. aethiopica*). *Trypanoasoma brucei* was considered as an out-group branch close to *Crithidia melifaca* in New World leishmaniasis (Fig. 3).

## Discussion

One of the current problematic issues in control programs and entomological monitoring of *Leishmania* infections is indiscrimination of low parasitic loads by PCR technique (12, 14). The rapid mass screening of infected sand fly population is provided valuable information in order to surveillance of infection controls, estimating the accurate prevalence of captured sand fly and transmission pattern of *Leishmania* parasite in endemic areas. In this exploration, we conducted a highly sensitive LAMP assay to identify the rapid mass screening of Old World leishmaniasis from captured sand flies in under field regions of southwest Iran.

The recorded analytical sensitivity of 0.01 *Leishmania* DNA (for infected sand flies) to 10^1^ promastigotes (for *L. major* in vitro culture) indicates the LAMP assay, not only is effectually identified the low loads of infected sand flies in resource-limited endemic regions but also it may be potentially recruited in low amastigote burden at classical and non-classical lesions.

In comparison to LAMP findings, the detection limit of PCR was 10^1^ *Leishmania* DNA for each infected sand fly. In addition, 10^4–6^ promastigotes were identified for *L. major* culture by PCR. This shows that PCR has less diagnostic sensitivity than LAMP assay in under field molecular investigations and clinical monitoring training. Theses sensitivity discrepancies among mentioned methods are described by some facts: first, activity of Taq DNA polymerase during PCR amplification is inhibited by tissue/blood components of sand flies such as myoglobin and protoporphyrin (12, 14–16) while, the *Bst* 2.0 DNA polymerase to overcome potential inhibitors in crude sand fly templates during LAMP amplification. Second, in a low *Leishmania* burden, a considerable amount of parasite DNA is lost during the extraction and purification processes that in this case, a set of PCR primers cannot be specifically annealed and amplified target templates (19).

Up to now, several *Leishmania*-DNA-based PCR methods including Nested-PCR, RFLP, and real-time PCR have been extensively used to identify the natural infection rates in sand flies (9–13). However, the mentioned assays take several hours with various diagnostic values (efficiency, sensitivity, and specificity) also are needed to specialized equipment (thermal cycler and electrophoresis system) which make their use unfeasible in resource-limited countries and under field conditions. In this study, the LAMP method detected the *Leishmania* spp. in shortest time possible (60 min in a heating block) without the need for a thermal cycler and electrophoresis system. In a similar study, the LAMP method employed for *Leishmania*-infected *Lutzomyia* recognition (Andean areas of Ecuador) using activity of wild-type *Bst* DNA polymerase (24). In this study, *Bst* 2.0 DNA polymerase was selected and tested on DNA replication of *Leishmania*-infected *Sergentomyia* and *Phlebotomus* sand flies. Evidence are shown that *Bst* 2.0 DNA polymerase displays more improved amplification speed, yield, salt tolerance and thermo-stability than *Bst* DNA polymerase.

In the current study, the LAMP as a field diagnostic tool was validated with 150 wild-collected sand flies. The rapid mass screening of sand flies from the 11 endemic areas resulted in the detection of ten *Leishmania* DNA-positive sand flies. The ten positives were confirmed to be *Leishmania* DNA by direct sequencing of the LAMP products. *Leishmania* DNA-positive sand flies were certainly infected with *L. major*.

The diagnostic sensitivity of LAMP technique was similar to nested-PCR (detection limit of 1 parasite in 1 ml of peripheral blood) for infected dog blood samples of VL (23).

A number of studies have been reported the identification of *Leishmania* infection by LAMP assay in sand flies, clinical samples and animal reservoirs (24, 27–29). The field applicability of the colorimetric Malachite green-based LAMP was shown assay as a field-friendly molecular tool for identification of *Leishmania*-infected *Lutzomyia* in the endemic areas of Ecuador (24). The reverse transcriptase LAMP was shown as a sensitive assay in detection of *Leishmania* parasites in both CL and visceral leishmaniasis (28). Another study demonstrated the LAMP combined with an FTA card as a rapid molecular diagnosis of CL patients (29). In this exploration, the occur-rence of non-specific figments due to laboratory contaminations and not to be affordable of *Bst* 2.0 DNA polymerase were addressed as the principal challenges of LAMP assay in our experiment.

## Conclusion

The current LAMP technique was able to amplify the all *Leishmania* spp. in one tube reaction within the shortest time possible using heating block /normal water bath. This method because of robustness, more sensitivity, lack of requirement of complicated equipment and visual discriminatory of positivity based on the turbidity of reaction mixture can be a promising alternative instead of single round PCR to identify low *Leishmania* loads and molecular epidemiological studies of leishmaniasis in resource-limited endemic of the world.
